# HBV infection may sensitize patients to alcoholic steatohepatitis and non-alcoholic steatohepatitis

**DOI:** 10.1093/gastro/goae018

**Published:** 2024-03-28

**Authors:** Hua Wang

**Affiliations:** Department of Oncology, The First Affiliated Hospital of Anhui Medical University, Hefei, Anhui, P. R. China; Inflammation and Immune Mediated Diseases Laboratory of Anhui Province, Anhui Medical University, Hefei, Anhui, P. R. China

Hepatitis B virus (HBV) infection remains one of the major common infectious diseases affecting the global population. Individuals with chronic hepatitis B (CHB) are at high risk of cirrhosis, acute liver failure, and hepatocellular carcinoma (HCC). Alcoholic steatohepatitis (ASH) is another global illness with high morbidity and mortality. In recent years, with the increasing incidence of CHB combined with ASH, the clinical of the co-occurrence of these two common liver disease etiologies remains a matter of debate and a more in-depth exploration of the interaction of CHB and ASH is required.

In the issue, Zhou *et al*. [1] carried out a comprehensive study to investigate the interaction between HBV infection and ASH. For this purpose, the authors used primary hepatocytes and cell lines, animal models, and clinical samples to explore this interaction in depth. In the first series of experiments, they revealed that the hepatitis B virus X protein (HBx) played a significant role in exacerbating alcohol-induced steatohepatitis, oxidative stress, and lipid peroxidation in mice. Next, metabolomic analysis further identified elevated levels of acetaldehyde in the serum and liver of HBx transgenic mice.

Most notably, the researchers discovered that HBx binds to mitochondrial aldehyde dehydrogenase 2 (ALDH2), resulting in the ubiquitin-dependent degradation of ALDH2. This mechanism provides an explanation for the detrimental interaction between HBV infection and ASH. These findings shed new light on why HBV-infected individuals are more susceptible to alcohol-induced liver disease and hold important implications for the management of patients with coexisting ASH and HBV infection.

The findings presented by Zhou *et al*. not only have implications for patients with both ASH and HBV infection but also extend to the understanding of non-alcoholic steatohepatitis (NASH). It has been noted that the histopathological characteristics of NASH are similar to those of ASH, with shared features such as fatty changes, lobular hepatitis, focal necrosis with mixed inflammatory infiltrates, Mallory bodies, and hepatocyte ballooning [2].

In an intriguing study, Baker and colleagues [3] reported elevated expression of genes responsible for alcohol metabolism in the livers of pediatric NASH patients. Subsequently, their group discovered elevated serum alcohol concentrations in pediatric NASH patients and identified altered gut microbiota as the source of increased endogenous alcohol production [4]. Furthermore, these researchers made similar observations of increased alcohol metabolism in the livers of adults with NASH [5]. These findings provided support for the alcohol hypothesis regarding the pathogenesis of NASH [6], and they have been substantiated by another study [7].

**Figure 1.**
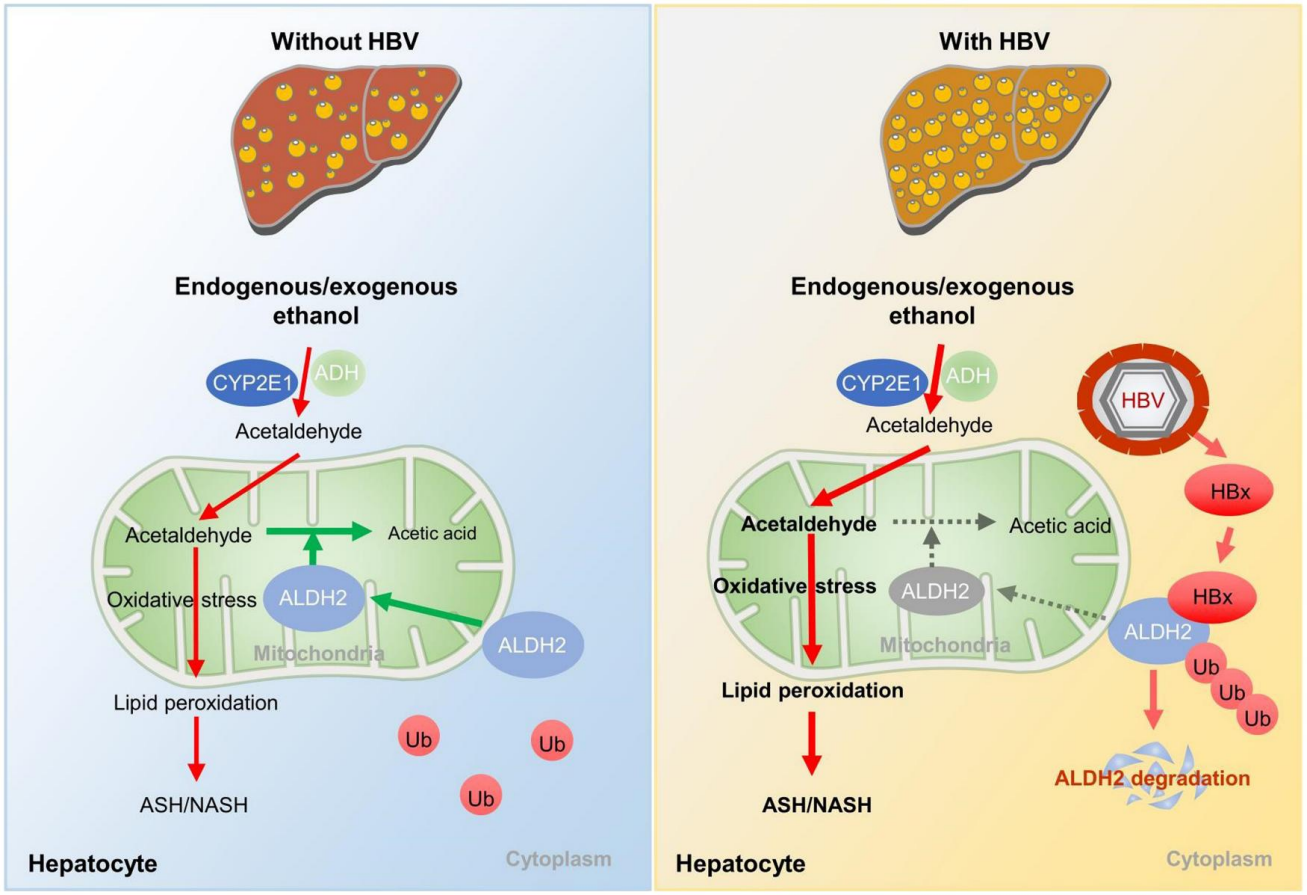
Schematic illustration of the interaction of HBV and ASH/NASH. *Notes*: In HBV-infected condition, HBx combined with mitochondrial ALDH2 and promoted ALDH2 ubiquitination and degradation, thereby induced acetaldehyde accumulation. Excessive acetaldehyde induced oxidative stress and lipid peroxidation aggravate ASH/NASH. HBV, hepatitis B virus; ASH, alcoholic steatohepatitis; NASH, non-alcoholic steatohepatitis; HBx, hepatitis B virus X protein; ALDH2, aldehyde dehydrogenase 2.

Therefore, it can be inferred that elevated alcohol metabolism, which necessitates sufficient ALDH2 activity to detoxify acetaldehyde and other toxic aldehydes into nontoxic aldehydes, is a shared characteristic of both ASH and NASH. Recent studies have identified the protective role of ALDH2 in experimental NASH in mice [8, 9]. Consequently, the degradation of ALDH2 caused by HBx may render patients more susceptible to both ASH and NASH (Figure 1).
